# A Systematic Multidisciplinary Process for User Engagement and Sensor Evaluation: Development of a Digital Toolkit for Assessment of Movement in Children With Cerebral Palsy

**DOI:** 10.3389/fdgth.2021.692112

**Published:** 2021-06-24

**Authors:** Lisa Kent, Ian Cleland, Catherine Saunders, Andrew Ennis, Laura Finney, Claire Kerr

**Affiliations:** ^1^School of Nursing and Midwifery, Queen's University Belfast, Belfast, United Kingdom; ^2^School of Computing, Ulster University, Newtownabbey, United Kingdom; ^3^Sunrise Medical, Lisburn, United Kingdom

**Keywords:** cerebral palsy, sensor technologies, user engagement, movement, multidiscipinary approach

## Abstract

**Objectives:** To describe and critique a systematic multidisciplinary approach to user engagement, and selection and evaluation of sensor technologies for development of a sensor-based Digital Toolkit for assessment of movement in children with cerebral palsy (CP).

**Methods:** A sequential process was employed comprising three steps: Step 1: define user requirements, by identifying domains of interest; Step 2: map domains of interest to potential sensor technologies; and Step 3: evaluate and select appropriate sensors to be incorporated into the Digital Toolkit. The process employed a combination of principles from frameworks based in either healthcare or technology design.

**Results:** A broad range of domains were ranked as important by clinicians, patients and families, and industry users. These directly informed the device selection and evaluation process that resulted in three sensor-based technologies being agreed for inclusion in the Digital Toolkit, for use in a future research study.

**Conclusion:** This report demonstrates a systematic approach to user engagement and device selection and evaluation during the development of a sensor-based solution to a healthcare problem. It also provides a narrative on the benefits of employing a multidisciplinary approach throughout the process. This work uses previous frameworks for evaluating sensor technologies and expands on the methods used for user engagement.

## Overview

Digital health technologies represent a new means of addressing modern healthcare challenges (1). For example, wearable sensor technologies can generate in-depth physiological and performance measurements outside of the laboratory environment, thereby, providing insight into real-world user behaviours. This can help patients and clinicians evaluate therapies and monitor progress over time. When thoughtfully developed and implemented, digital solutions can augment the role of the health care professional and enhance patient participation in health care decisions.

The following report describes a systematic multidisciplinary approach to user engagement, and selection and evaluation of sensor technologies for development of a sensor-based Digital Toolkit for assessment of movement in children with cerebral palsy (CP).

## Background

Cerebral palsy (CP) is a neurodevelopmental condition caused by injury to the developing brain. It is the most frequent cause of physical disability among children and has a life-long impact on the individual and their use of healthcare (2, 3). People with CP experience disordered posture and movement that in turn causes limitations in activities (e.g., walking) (4, 5). This can lead to physical inactivity, increased risk of developing cardiovascular disease, and limited participation and social integration in the home and community (3, 6, 7).

Rehabilitative and assistive devices, such as mobility and postural aids, can help restore or replace the loss of activity caused by a disability such as CP (8). However, a large proportion of interventions for children with CP have low or inconclusive evidence supporting their effectiveness (9, 10). As assistive devices (such as wheelchairs, walking frames, and communication devices) form a large part of standard care for children with CP, a systematic, objective, and disciplined approach to measuring clinical outcome is needed when prescribing them (10). To date, specialised equipment and technology have been vastly under-researched. This is potentially due to benefits, such as independent mobility, being easily observable yet difficult to objectively quantify outside of the clinic or laboratory environment. Some simple clinical tests allow for a quick overview of a patient's condition, but do not afford more in-depth evaluation of individual impairments or components of activities that may, or may not, be amenable to change. Nevertheless, considering device abandonment issues and associated costs, extensive efficacy research is warranted at both an individual and a population level. Moreover, prescribing assistive technology may raise expectations of good outcomes by the patient and family and give rise to an overinflated perception of high-quality expert care. Thus, it is essential to know if the interventions are working, to prevent device abandonment, false hopes, and unnecessary effort (10). In addition, The Royal Academy of Engineering has called for improved methodologies to obtain evidence for not only safety and performance of medical devices but also for efficacy (11).

To this end, a Digital Toolkit of sensor-based technologies is currently being developed for assessment of movement in children with CP, in relation to rehabilitative and assistive devices. The Digital Toolkit will provide a means of integrating and visualising data from commercially available, wearable sensors to provide a platform for data-driven real-world evaluation of effect of physical therapies, and rehabilitative and assistive devices. To meet the needs of users and provide valuable insights, a process of user engagement and device evaluation has been used to inform the development of the Digital Toolkit.

Engagement with users (e.g., industry, clinicians, patients, and families) throughout the technology development pipeline is essential in ensuring that a product that adds significant value is translated into practice and ultimately delivers impact (12, 13). There is no universally accepted framework for engaging with users in the development of digital health technologies. However, in health and social care research in the UK, meaningful involvement of users through Patient, and Public Involvement (PPI) has increased in recent years and is now well-established (14, 15). From a technology perspective, two research groups have recently proposed processes for user engagement and technology selection and evaluation (12, 16). Further to this, there are established principles for user-centred design [ISO 9241-210:2019(en)], however, these tend to focus on how a user engages with computer-based technology and have limited value in guiding selection of sensors for digital health (17).

It is good practice to disseminate and critically reflect on the approaches used for user engagement in healthcare. Principles of the Short Form Guidance for Reporting Involvement of Patients and the Public checklist (GRIPP2), developed to improve reporting of patient and public involvement (PPI) in research, have been embedded across this report (18).

The aim of this report is to document and critique a systematic multidisciplinary approach to user engagement, and selection and evaluation of sensor technologies for development of a sensor-based Digital Toolkit for assessment of movement in children with cerebral palsy (CP).

## Materials and Methods

A sequential process was employed comprising three steps:

Step 1: Define user requirements, by identifying domains of interest;
Step 2: Map domains of interest to potential sensor technologies; and
Step 3: Evaluate and select appropriate sensors to be incorporated into the Digital Toolkit.

The process employed a combination of principles from frameworks based in either healthcare or technology design (12, 14, 16).

Scope of the project: Inform the development of a Digital Toolkit to aid in-depth objective evaluation of effectiveness of mobility devices in children with CP.

### Step 1: Define User Requirements

#### 1a. Identification of Potential Domains of Interest

The Core Set for CP, derived from the World Health Organisation's International Classification of Functioning, Disability and Health Children and Youth Version - ICF-CY (WHO 2007), was used to identify potential domains of interest to target with sensor technologies (19, 20). The ICF-CY is a framework for describing and organising information on functioning and disability. The framework covers body structures (anatomical parts of the body such as organs, limbs and their components) and functions (physiological functions of body systems), and activity (the execution of a task or action by an individual) and participation (involvement in a life situation). The Core Set for CP is a list of 135 ICF-CY domains that are considered most important for describing the functional profile of individuals with CP aged 0 to 18 years. Through discussion, the research team compiled a short-list of domains from the Core Set which were of potential relevance to the Digital Toolkit (Table 1). Whilst the aim of the Digital Toolkit is to aid assessment of movement in children with CP in relation to rehabilitative and assistive devices, it was agreed that the current project would focus on mobility devices. Selection criteria were based on the research team's consideration of the potential of the ICF-CY domain to either:

a) demonstrate an immediate change, i.e., a change that may be observed *during* use of a mobility device;
b) demonstrate a change in response to *sustained use*, i.e., a change that may be observed when the device is used as part of a training program over a period of time; or
c) domains that may have a bearing on the child's ability to use a mobility device.

**Table 1:** Short list of domains of interest compiled from ICF-CY core set for CP.

**ICF-CY Category**	**Definition**
**Impairment–body functions**	
B280 sensation of pain	Sensation of unpleasant feeling indicating potential or actual damage to some body structure. Inclusions: sensations of generalized or localized pain, in one or more body part, pain in a dermatome, stabbing pain, burning pain, dull pain, aching pain; impairments such as myalgia, analgesia and hyperalgesia.
B440 respiratory functions	Functions of inhaling air into the lungs, the exchange of gases between air and blood, and exhaling air. Inclusions: functions of respiration rate, rhythm and depth; impairments such as apnoea, hyperventilation, irregular respiration, paradoxical respiration, and brochial spasm, and as in pulmonary emphysema; upper pulmonary obstruction, reduction in airflow through upper and lower airways. Exclusions: respiratory muscle functions (b445); additional respiratory functions (b450); exercise tolerance functions (b455).
B455 exercise tolerance functions	Functions related to respiratory and cardiovascular capacity as required for enduring physical exertion. Inclusions: functions of physical endurance, aerobic capacity, stamina, and fatigability. Exclusions: functions of the cardiovascular system (b410–b429); haematological system functions (b430); respiration functions (b440); respiratory muscle functions (b445); additional respiratory functions (b450).
B530 weight maintenance functions	Functions of maintaining appropriate body weight, including weight gain during the developmental period. Inclusions: functions of maintenance of acceptable Body Mass Index (BMI); and impairments such as underweight, cachexia, wasting, overweight, emaciation, and such as in primary and secondary obesity. Exclusions: assimilation functions (b520); general metabolic functions (b540); endocrine gland functions (b555).
B710 mobility of joint functions	Functions of the range and ease of movement of a joint. Inclusions: functions of mobility of single or several joints, vertebral, shoulder, elbow, wrist, hip, knee, ankle, small joints of hands, and feet; mobility of joints generalized; impairments such as in hypermobility of joints, frozen joints, frozen shoulder, arthritis. Exclusions: stability of joint functions (b715); control of voluntary movement functions (b760).
B730 muscle power functions	Functions related to the force generated by the contraction of a muscle or muscle groups. Inclusions: functions associated with the power of specific muscles and muscle groups, muscles of one limb, one side of the body, the lower half of the body, all limbs, the trunk and the body as a whole; impairments such as weakness of small muscles in feet and hands, muscle paresis, muscle paralysis, monoplegia, hemiplegia, paraplegia, quadriplegia, and akinetic mutism. Exclusions: functions of structures adjoining the eye (b215); muscle tone functions (b735); muscle endurance functions (b740).
B7350 muscle tone functions	Functions related to the tension present in the resting muscles and the resistance offered when trying to move the muscles passively. Inclusions: functions associated with the tension of isolated muscles and muscle groups, muscles of one limb, one side of the body and the lower half of the body, muscles of all limbs, muscles of the trunk, and all muscles of the body; impairments such as hypotonia, hypertonia and muscle spasticity, myotonia, and paramyotonia. Exclusions: muscle power functions (b730); muscle endurance functions (b740).
B740 muscle endurance functions	Functions related to sustaining muscle contraction for the required period of time. Inclusions: functions associated with sustaining muscle contraction for isolated muscles and muscle groups, and all muscles of the body; impairments such as in myasthenia gravis. Exclusions: exercise tolerance functions (b455); muscle power functions (b730); muscle tone functions (b735).
B760 control of voluntary movement functions	Functions associated with control over and coordination of voluntary movements. Inclusions: functions of control of simple voluntary movements and of complex voluntary movements, coordination of voluntary movements, supportive functions of arm or leg, right left motor coordination, eye hand coordination, eye foot coordination; impairments such as control and coordination problems, e.g., clumsiness and dysdiadochokinesia. Exclusions: muscle power functions (b730); involuntary movement functions (b765); gait pattern functions (b770).
B765 involuntary movement functions	Functions of unintentional, non- or semi-purposive involuntary contractions of a muscle or group of muscles. Inclusions: involuntary contractions of muscles; impairments such as tremors, tics, mannerisms, stereotypies, motor perseveration, chorea, athetosis, vocal tics, dystonic movements, and dyskinesia. Exclusions: control of voluntary movement functions (b760); gait pattern functions (b770).
B770 gait pattern functions	Functions of movement patterns associated with walking, running, or other whole body movements. Inclusions: walking patterns and running patterns; impairments such as spastic gait, hemiplegic gait, paraplegic gait, asymmetric gait, limping, and stiff gait pattern. Exclusions: muscle power functions (b730); muscle tone functions (b735); control of voluntary movement functions (b760); involuntary movement functions (b765).
**Activity and participation**	
D410 changing basic body position	Getting into and out of a body position and moving from one location to another, such as rolling from one side to the other, sitting, standing, getting up out of a chair to lie down on a bed, and getting into and out of positions of kneeling or squatting. Inclusion: changing body position from lying down, from squatting or kneeling, from sitting or standing, bending and shifting the body's centre of gravity. Exclusion: transferring oneself (d420).
D415 maintaining a body position	Staying in the same body position as required, such as remaining seated or remaining standing for work or school. Inclusions: maintaining a lying, squatting, kneeling, sitting, and standing position.
D420 transferring oneself	Moving from one surface to another, such as sliding along a bench or moving from a bed to a chair, without changing body position. Inclusions: transferring oneself while sitting or lying. Exclusion: changing basic body position (d410).
D435 moving objects with lower extremities	Performing coordinated actions aimed at moving an object by using the legs and feet, such as kicking a ball or pushing pedals on a bicycle. Inclusions: pushing with lower extremities; kicking.
D450 walking (d4500 walking short distances)	Moving along a surface on foot, step by step, so that one foot is always on the ground, such as when strolling, sauntering, walking forwards, backwards, or sideways. (d4500 = Walking for less than a kilometre, such as walking around rooms or hallways, within a building or for short distances outside.) Inclusions: walking short or long distances; walking on different surfaces; walking around obstacles. Exclusions: transferring oneself (d420); moving around (d455).
D455 moving around	Moving the whole body from one place to another by means other than walking, such as climbing over a rock or running down a street, skipping, scampering, jumping, somersaulting, or running around obstacles. Inclusions: crawling, climbing, running, jogging, jumping, swimming, scooting, rolling, and shuffling. Exclusions: transferring oneself (d420); walking (d450).
D460 moving around in different locations	Walking and moving around in various places and situations, such as walking between rooms in a house, within a building, or down the street of a town. Inclusions: moving around within the home, crawling or climbing within the home; walking or moving within buildings other than the home, and outside the home and other buildings.
D465 moving around using equipment	Moving the whole body from place to place, on any surface or space, by using specific devices designed to facilitate moving or create other ways of moving around, such as with skates, skis, scuba equipment, swim fins, or moving down the street in a wheelchair or a walker. Exclusions: transferring oneself (d420); walking (d450); moving around (d455); using transportation (d470); driving (d475).
**Environment and personal**	
e115 products and technology for personal use in daily living	Equipment, products and technologies used by people in daily activities, including those adapted or specially designed, located in, on or near the person using them. Inclusions: general and assistive products and technology for personal use. Exclusions: products and technology for personal indoor and outdoor mobility and transportation (e120); products and technology for communication (e125).
e120 products and technology for personal indoor and outdoor mobility and transportation	Equipment, products and technologies used by people in activities of moving inside and outside buildings, including those adapted or specially designed, located in, on or near the person using them. Inclusions: general and assistive products and technology for personal indoor and outdoor mobility and transportation.
e140 products and technology for culture, recreation and sport	Equipment, products and technology used for the conduct and enhancement of cultural, recreational and sporting activities, including those adapted or specially designed. Inclusions: general and assistive products and technology for culture, recreation and sport. Exclusion: products and technology for play (e1152).
**Additional**	
Health related quality of life	Health-related quality of life (HRQoL) is a multi-dimensional concept that includes domains related to physical, mental, emotional, and social functioning. It goes beyond direct measures of population health, life expectancy, and causes of death, and focuses on the impact health status has on quality of life. NB A related concept of HRQoL is well-being, which assesses the positive aspects of a person's life, such as positive emotions and life satisfaction. See also Karimi and Brazier 2016: https://link.springer.com/article/10.1007%2Fs40273-016-0389-9.
Self-concept	An idea of the self, constructed from the beliefs one holds about oneself and the responses of others; confidence in one's own worth or abilities; self-respect.
Perceptions of effort/ease of caregiving	Level of difficulty in assisting child to perform self-care activities safely and fulfil the child's physical needs in a confident and competent manner within reasonable expectations/Caregiver's perception of burden of care.

The short-listed domains and their ICF-CY standard definitions were compiled. To aid communication with non-clinical users, “lay” definitions were developed by the research team.

#### 1b. Rapid Review

A list of outcome measures reported as being used in children with CP and relevant to each ICF-CY short-listed domain, was generated through a series of rapid literature reviews. Each review used key words elicited from the ICF-CY domain definitions. Medline and Embase were searched from inception using key words specific to each domain. For example, for the domain “B455 Exercise Tolerance Functions” the following key terms were used: (“exercise tolerance” OR “respiratory capacity” OR “cardiovascular capacity” OR “physical endurance” OR “aerobic capacity” OR “stamina” OR “fatigability” OR “fitness” OR “exercise test” OR “cardiorespiratory” OR “cardiopulmonary”) AND “cerebral palsy.”

A matrix was produced detailing included ICF-CY domains (code, category name, sub-domains, standard definitions), measurement instruments used in clinical practice or research in CP for each domain, any reported technological/sensor-based measurement solutions, references to relevant literature on clinimetric properties, and links to websites where instruments could be obtained.

#### 1c. User Engagement

The domains and their associated definitions, short-listed in **Step 1a**, were presented in separate deliberative workshops to three stakeholder groups (21). The stakeholder groups were: (i) children with CP and their families; (ii) clinicians; and (iii) industry. The workshops were facilitated by both the clinical and computer science researchers. The team adhered to the principles of patient and public involvement (PPI) as proposed by NIHR (14). The aim of the workshops was to obtain a broad range of perspectives from end-users on the relative importance of the short-listed domains.

##### Workshops With Children and Families

Two deliberative workshops were held with families of children with CP. Workshops were advertised via social media (Twitter and Facebook) and using two community mailing lists (Northern Ireland Cerebral Palsy Register Community Mailing List, James Leckey Design Families Communications Email List). Two workshops were convened at a central location with accessible parking at different times (one afternoon, one evening) in an effort to accommodate work, childcare, and access arrangements. Travel expenses of workshop participants were reimbursed and a gift card to the value of £20 was provided in acknowledgement of their time and contributions.

At each workshop the research team explained the project to attendees: an overview of the short-listed domains and rapid review (described above in **Steps 1a and 1b**) was presented and a video demonstration of a range of potential sensors was shown. Following group discussion, each adult was provided with printed copies of the short-listed domains and their lay definitions and was asked to rank order the domains from most (ranked 1) to least important, in their opinion, with respect to measuring the effects of mobility devices. Discussions were not audio-recorded but researchers took anonymised notes during the workshop. Attendee rank order lists were collated at the end of each workshop.

##### Workshop With Healthcare Professionals

A deliberative workshop was held for physiotherapists and technical instructors involved in the care of children with CP in school or community settings. After a short presentation outlining the findings of the rapid review (**Step 1b)**, attendees were provided with the short-list of ICF-CY domains and definitions and asked to rank the domains in order of priority (1 being highest priority) without referring to others in the group. The four highest ranked domains were presented back to the group and time allowed for discussion of the results. The group were then asked to consider the measurement instruments (highlighted by the rapid review in **Step 1b**) associated with the four highest ranked domains and asked to confirm if they were aware of the instruments, were they currently available to them, and had they used them. They were also asked for their opinions on whether the instrument would be useful in a research environment and/or clinical practice. The group was also asked if they were aware of any alternative potential measurement instruments or technology solutions.

##### Workshop With Industry

The research team met with industry specialists (designers, engineers, research and development management, and therapists involved in manufacture of supportive equipment for children with CP) to present the findings of the ICF-CY domain short-listing and rapid review (**Steps 1a and 1b**). The short-listed domains were iteratively discussed and industry priorities in relation to domains of interest were noted.

#### 1d. Comparison and Agreement of User Requirements

The four highest ranked domains from the family and healthcare profession stakeholder groups were compared and integrated with the findings from the industry workshop. Final domains of interest across stakeholder groups were agreed within the research team. Workshop attendees were provided with a written summary of findings and the opportunity to provide further feedback.

### Step 2: Device Search

The prioritised domains from **Step 1** were systematically mapped to feasible sensor technological solutions based on the process proposed by Caulfield et al. (12). To achieve this, two researchers independently searched online using key terms related to each domain. Initially, researchers sought to identify technology-based solutions based on the following criteria:

i) is the system commercially available,
ii) is the solution portable and could it be used in a community setting,
iii) does the system allow for integration i.e., access to data in its rawest form/access to the device through an application programming interface (API) or software development kit (SDK), and
iv) has the device been used with children with CP or other movement disorders.

Whilst non-contact wireless solutions show promise in monitoring of vital signs, they currently suffer from a number of technical challenges limiting their use in clinical and community settings. That, coupled with a lack of a commercially available solution meant that they were not considered for inclusion in the Digital Toolkit. Further, for domains that could not be mapped to objective technology-based solutions (e.g., health-related quality of life), the team identified alternative validated tools, such as questionnaires, that could be used to capture these elements.

### Step 3: Device Evaluation

Once relevant solutions were identified, researchers undertook a detailed technical evaluation to ensure the technologies could be successfully integrated. Criteria for consideration were based on a combination of those proposed by Caulfield et al. (12) and Booth et al. (16) and are presented in Supplementary Material. The technical evaluation expanded on the criteria specified above (**Step 2**, Device Selection) to further refine the selection process. Additional information assessed at this device evaluation step included device dimensions, sensors included, battery life, connectivity requirements and data access, as well as the availability of established software for data collection and interpretation. The team also investigated developer support for integration such as the availability of an SDK or API. Information on regulatory compliance and scientific evidence, specifically, if the device had been clinically validated and if it had been used in a CP or paediatric population was sought. Additionally, purchase information such as device availability in the target country, cost, and the availability of technical support was determined. Finally, human factors such as comfort and usability, as well as the availability of sizes suitable for a paediatric population, were considered. Essentially, we sought to identify established commercial systems that had been validated in the population and setting of interest, that allowed integration and had established software for analysis. Researchers searched online repositories (IEEE Explore, Web of Science and Google Scholar) for device names along with search terms such as “children,” “youth,” “paediatric,” and “cerebral palsy” to identify validation studies and studies reporting use of the with the target population.

## Results

### Step 1: Define User Requirements

#### 1a. Identification of Potential Domains of Interest

Eleven impairment and eight activity domains that were likely to be amenable to change through use of a mobility device were shortlisted by the research team (Table 1). Three additional domains were also identified that are not included in the ICF-CY Core Set for CP but would potentially be amenable to change through use of a mobility device: health related quality of life, self-concept, and ease of care-giving. Whilst these domains are not included in the ICF-CY or core sets, they are referred to within the ICF-CY section “future directions for ICF.”

#### 1b. Rapid Review

No sensor-based technological measurement solutions were identified from the literature retrieved. For example, the ICF-CY domain “B455 Exercise Tolerance Functions” mapped to various “low-tech” solutions such as timed walk tests (1 minute walk test, 2 minute walk test, 6 minute walk test), set-distance walking test (10 m walk test), shuttle tests (10 m shuttle test, 10 × 5 sprint test) and “high-tech” laboratory tests using online gas analysis.

#### 1c. User Engagement

##### Workshops With Children and Families

Sixteen families responded to the initial advert, however, due to family and work commitments nine families were unable to attend the scheduled workshops. Seven families (nine adults in total) thus attended one of two scheduled family workshops. In total three grandparents and six parents of children ranging in age from <2 to 15 years attended. Four children also attended with their families. Mean score and rank for each domain is presented in Table 2. Of the 11 short-listed ICF-CY impairment (body function) domains, families ranked the following as most important: (1) muscle tone functions, (2) sensation of pain, (3) mobility of joint functions, and (4) muscle power functions. Of the eight short-listed ICF-CY activity and participation domains, families ranked (1) maintaining a body position, (2) moving around using equipment, (3) walking (short distances), and (4) moving objects with lower extremities as the most important. In addition, the groups unanimously agreed that it was important to consider health-related quality of life, self-esteem and ease of care when evaluating movement in children with CP.

**Table 2:** Results of workshops.

	**Family workshops**		**Clinician workshop**	
	**Mean score**	**Rank**	**Mean score**	**Rank**
**Impairment–body functions**				
Sensation of pain	4.11	2	4.57	3
Respiratory functions	8.38	10	6.29	8
Exercise tolerance functions	7.22	9	3.71	1
Weight maintenance functions	9.89	11	10.21	11
Mobility of joint functions	4.33	3	4.14	2
Muscle power functions	4.78	4	5.36	5
Muscle tone functions	2.56	1	5.93	7
Muscle endurance functions	5.56	6	6.71	9
Control of voluntary movement functions	5.22	5	5.21	4
Involuntary movement functions	7.11	8	8.57	10
Gait pattern functions	6.56	7	5.54	6
**Activity and participation**				
Changing basic body position	4.50	5	2.00	1
Maintaining a body position	3.44	1	4.92	6
Transferring oneself	6.38	8	4.00	3
Moving objects with lower extremities	4.44	4	7.08	8
Walking (short distances)	4.00	3	5.31	7
Moving around	4.56	6	4.46	4
Moving around in different locations	4.56	6	4.46	4
Moving around using equipment	3.38	2	3.69	2

*Highlighted cells = Ranked in top 4*.

##### Workshop With Healthcare Professionals

Fourteen clinicians with a wide range of experience were present at the workshop (11 physiotherapists and three technical instructors). The following four ICF-CY impairment (body function) domains, of the eleven short-listed, were ranked as most important by clinicians: (1) exercise tolerance functions, (2) mobility of joint functions, (3) sensation of pain, (4) control of voluntary movement functions. The top ranked ICF-CY activity and participation domains, of the eight short-listed, were: (1) changing basic body position, (2) moving around using equipment, (3) transferring oneself, and in joint fourth, moving around, and moving around in different locations. All healthcare professionals agreed that it was important to consider health-related quality of life, self-esteem and ease of care when evaluating movement in children with CP.

With regards to knowledge and use of measurement instruments related to the top ranked ICF-CY impairment (body function) domains, healthcare professionals were most familiar with the 6 minute walk test (exercise tolerance), goniometry (mobility of joint) and visual analogue scales (pain). The group considered that these instruments were useful in both clinical and research settings.

For measurement of control of voluntary movement, a small number of healthcare professionals had an appreciation of laboratory-based measurements such as electromyography and kinematic measures, however, use of these in their area was limited. Instead, a subjective description of control of voluntary movement was reported to be the norm in clinical practice in community healthcare settings.

No additional instruments or sensor-based technological solutions were suggested for any of the domains identified as important.

##### Workshop With Industry

A workshop was held with four industry specialists working in a commercial enterprise concerned with design and manufacture of mobility device products. Professional backgrounds of participants were therapy, biomedical engineering and research and development. The priorities of the industry workshop participants were primarily focused on physiological measures aligned with the ICF-CY impairment level, such as discerning muscle activation patterns and ascertaining the “smoothness” of movement. No additional domains of interest were identified by industry specialists.

#### 1d. Comparison and Agreement of User Requirements

The engagement process identified similarities and differences between groups of stakeholders regarding domains they considered important to measure. Two top-ranked ICF-CY impairment domains were common to both families and clinicians: mobility of joint functions and sensation of pain. Families' top-ranked ICF-CY impairment domain was muscle tone, which was ranked 7th (of 11) by clinicians, whereas, clinicians' top-ranked domain was exercise tolerance, which families ranked 9th (of 11). Differences in perceived priorities for measurement between families and clinicians were also evident for the ICF-CY activity and participation domains, as shown in Table 2: only “moving around using equipment” was common to both respondent groups. Strong agreement between clinicians and families was evident in relation to the importance of health-related quality of life, ease of care, and self-concept. Domains rated least important were broadly consistent between stakeholder groups and included weight maintenance, respiratory function, and involuntary movements. These domains were thus removed from subsequent steps in the development of the Digital Toolkit given their lower perceived importance.

A high-level summary of findings was communicated directly to all workshop attendees and circulated via the Northern Ireland Cerebral Palsy Community Mailing List to disseminate the findings of the workshops to the wider community. No further feedback or suggestions were received.

### Step 2: Device Search

The research team successfully mapped the prioritised clinical domains in **Step 1** (mobility of joint functions, muscle tone, muscle power, control of voluntary movement, exercise tolerance, and moving around using equipment), to potential instrumented measurements (Table 3), with the exception of the “sensation of pain” domain. Instrumented techniques commonly used to measure joint mobility, voluntary movement, muscle tone, muscle power and moving around, included three dimensional (3D) motion capture and surface electromyography (sEMG) (24). Methods to measure exercise tolerance included vital sign monitors for heart rate (HR) and respiration rate (RR) (23).

**Table 3:** Mapping of instrumented physiological measures from Step 3.

**Clinical domain**	**Underlying instrumented physiological measure**	**Identified measurement solution**
Sensation of pain	No instrumented method is available to assess Sensation of pain. A number of self-reported scales exist. A small number of papers have measured response of the sympathetic system to stimuli by measuring the changes in heart rate (HR), heart rate variability (HRV), pulse amplitude (PA), and galvanic skin response (GSR). Though, these are not relevant for the current experimentation (22).	None.
Exercise tolerance functions	In submaximal tests, like that which would be undertaken in a clinical setting, minute walking tests are common. This is due to their easy to administer and inexpensive nature (23). It has been suggested that in addition to distance covered, a measure of energy expenditure may be useful in differentiating between physical and physiological factors. For research studies, variables such metabolic energy (e.g., oxygen consumption) could be used. In the clinic, however, this is not usually feasible. It has therefore been recommended that heart rate should be monitored both at rest and during the walk when using the 6-MWT. Other factors that could be measured include heartrate variability, Exercise intensity level (% of heart rate reserve), heart rate reserve, breathing pattern, oxygen and carbon dioxide levels in each breath, work rate.	Wearable vital signs monitors: 1. Zypher bioharness 2. Hexoskin 3. Polar H10 4. Shimmer 3
Muscle power functions	Muscle torque and force measures were assessed by force sensors in both lower and upper extremities (24). For the lower extremities, as investigated in this research, maximum isometric flexion and extension torques of the knee are generally measured. A number of studies have previously reported the relationship between the surface EMG and muscle force (25, 26). Some authors have concluded that the magnitude of the EMG signal is directly proportional to muscle strength for isometric and/or isotonic contractions with constant speed for various muscles. Although, surface EMG is used to quantify muscle activity, the relationship between force, and surface EMG during voluntary contractions is not fully understood (27). EMG has been used to understand differences in muscle activity levels during daily activities between children with cerebral palsy (28).	Force sensors will not be used within the current study. EMG will be used to understand muscle activity.
Muscle tone functions	Muscle tone and motor flex is commonly measured using sEMG (24). This is either used alone or in combination with force sensors or position measures. These measurements are intended to distinguish between dyskinetic and spastic CP. They can also be used to determine the relation of muscle tone and motor reflex in dyskinetic CP and the influence of muscle tone and motor reflex on control of voluntary movement.	sEMG solutions: 1. Delsys trigno avanti 2. Noraxon ultium 3. Cometa wave 4. BTS engineering freeemg
Control of voluntary movement functions	Control of voluntary movement is generally assed by analyzing muscle activity through analysis of sEMG (24). The simultaneously contraction of two or more muscles around a joint and the relative contribution of muscle activity measured during a repetitive task are commonly used. This has however only been tested in small groups and requires further development to be used in a clinical setting.	
Mobility of joint functions	Position and joint angle measurements are typically performed using 3D motion tracking. This is sometimes combined with other measures such as electrogoniometry to assess spatiotemporal and kinematic parameters during different tasks (24).	Full body motion capture: 1. Xsens, MTx awinda 2. Nueron, perception 3. APDM, The opal 4. Qualisys 5. Vicon 6. GaitUp, Physiolog 5 7. Trivision

With the underlying instrumented measurements identified, the research team then began to search for suitable sensor technologies that met the requirements highlighted previously in **Step 2** of the Methods Section. Findings, by ICF-CY domain, are summarised below.

#### Exercise Tolerance

Wearable vital signs monitors have become increasingly common and accessible in recent years. Many commercial fitness and lifestyle monitors, such as Fitbit and Apple Watch, are now capable of recording information on a user's heart rate and activity. The research team chose to focus on chest worn vital signs monitors rather than wrist or patch-based monitors for two reasons. Firstly, heart rate measurements from chest strap-based monitors have been shown to be more accurate compared to optical wrist-based monitors (29). Secondly, a chest strap was deemed more practical for participants, particularly considering the locations of other sensors that the participant would be required to wear during the study. Chest based monitors come in two main formats, chest straps or garment integrated (30).

#### Joint Mobility and Moving Around

The device search identified motion capture (MoCap) as a useful tool for objectively quantifying human movement. It is often used with individuals with CP in a clinical or laboratory setting to analyse walking patterns (31). However, whilst optical based MoCap is considered the gold standard, most optical MoCap systems are costly, cumbersome, suffer from optical occlusion, and require a dedicated laboratory (31). More recently wearable MoCap systems based on inertial measurement unit (IMU) have come onto the market. These systems employ sensors fusion and provide information on degrees of freedom, force, and moment to estimate joint moments and powers.

#### Muscle Power, Muscle Tone, and Control of Voluntary Movement

Surface electromyography (sEMG) was identified as a non-invasive means of investigating and evaluating skeletal muscle activity. Contemporary sEMG systems consist of wireless solutions and generally come in garment-based or fully wireless sensor forms, thus meet our “portability” requirement (32). Garment-based solutions tend to focus on specific areas rather than targeted muscles and are aimed more at consumers rather than clinical research markets. A number of systems that have been used in clinical research were identified. These solutions allow for real-time feedback and can be used outside of a laboratory setting. Many of these solutions also incorporated IMU affording the potential for kinematic data capture in tandem with sEMG. Whilst typically more accurate, the clinical systems were considerably more expensive than consumer systems.

### Step 3: Device Evaluation

With a range of suitable systems identified from the device search in **Step 2**, the research team set out to technically evaluate each of the devices. Technical information was gleaned from manufacturer websites, with information on clinical validation and use of the device in the target population coming from academic publications. The results of the technical evaluation are summarised in Supplementary Material. With the explosion of wearable technologies now available on the market, there was ample choice of instrumented solutions available for review.

Vital signs monitors typically captured information on heart rate, respiration rate and activity. Some devices captured additional clinical information including heart rate variability and temperature. Devices ranged in price from approximately £200–£1,000. Battery life for these devices typically lasted at least 24 h and up to 65 h, depending on the sampling and data transmission rates. These devices usually communicated over standard transmission protocols such as Bluetooth and provided good access to raw data.

EMG solutions were generally the most expensive instrumented element of the Digital Toolkit. System prices ranged from £12,000 to £20,000. The price was typically associated with the substantial number of sensors required to measure the whole body (typically 16 sensors). Nodes generally included EMG in combination with other sensors to measure movement such as IMU. EMG systems were generally wireless, transmitting data over proprietary data transmission. Battery life was typically 6–10 h depending on the sampling rate and number of sensors.

For motion capture systems, there was a wide range of solutions available, from those that use one or two sensors to track specific measures of gait, to full body motion capture solutions that use up to 24 sensor nodes. These solutions typically incorporated IMU sensors with the option to add other sensing technologies such as force sensors, EMG, and electro-goniometers. Depending on the sampling rate and the number of sensors, battery life lasted between 3 and 18 h. Data was typically transmitted over Wi-Fi or proprietary wireless data transmission. Traditional camera-based systems were also available. However, these tended to be less portable, susceptible to occlusion and limited to a set measurement volume.

Across all three application areas, some of the solutions identified were targeted more toward consumer markets, rather than research. These solutions tended to be lower cost, however, are less likely to have been clinically validated. The most established market was that of vital signs monitoring. Devices in this category were of reasonable cost, all those identified had been clinically validated and many had been used with the target population. Motion capture systems are also very well-established with many solutions commercially available. Whilst the cost of these systems is still high, particularly for research focused systems, this is likely due to the technical complexity of these solutions which incorporate multiple sensors and require complex algorithms for data processing.

#### Summary of Results

**Figure 1:**
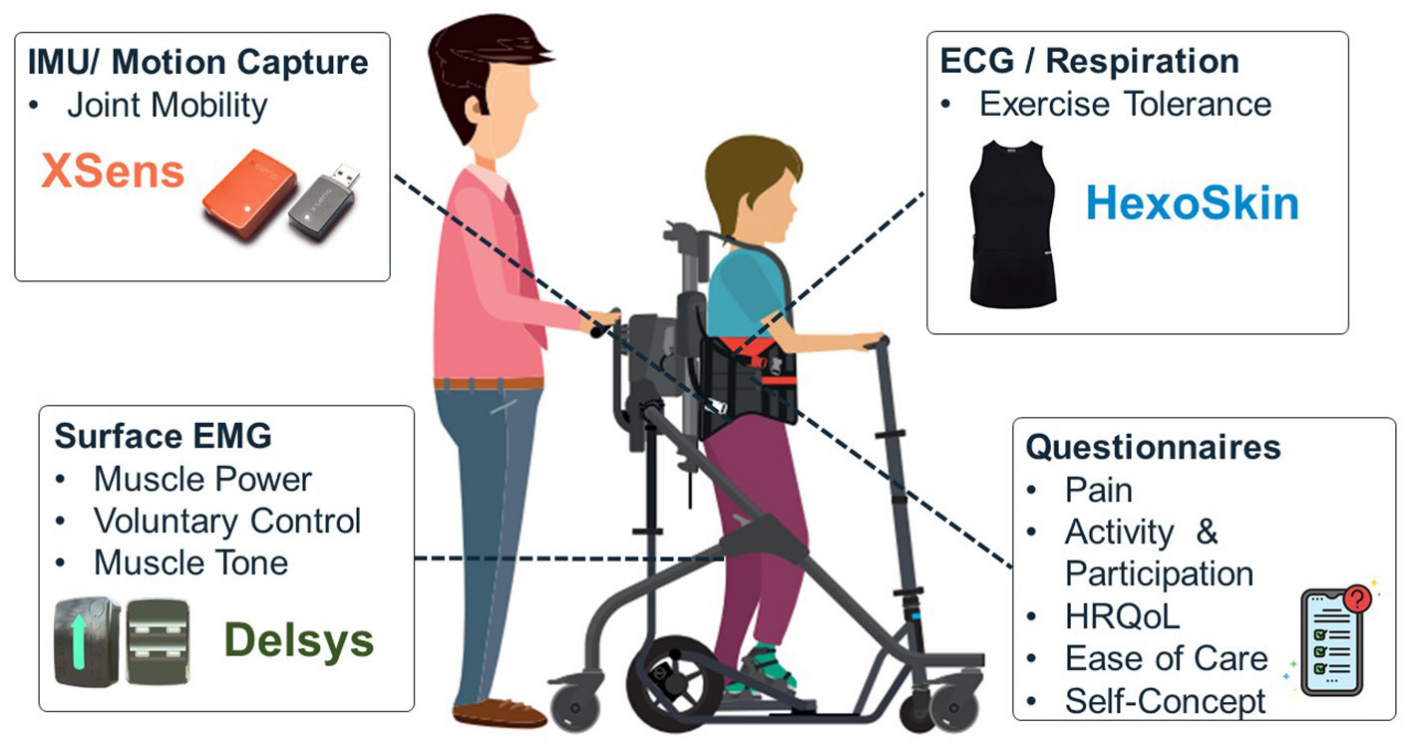
Overview of user-prioritised domains of interest and selected sensor technology.

Through combination of the aforementioned steps, the multidisciplinary team developed a Digital Toolkit consisting of IMU/ motion capture (Xsens) to evaluate joint mobility, ECG and Respiratory Inductive Plethysmography (Hexoskin) to evaluate exercise tolerance, and surface EMG (DELSYS) to explore muscle power, control of voluntary movement and muscle tone (see Figure 1).

Hexoskin was chosen as an exercise tolerance measurement solution, over other vital signs solutions, as the system met selection criteria in terms of measurement requirements (ECG, HR, and RR), interoperability, access to raw data and portability, and was clinically validated with a paediatric population. The Zephyr Bio Harness was considered an appropriate alternative. Nonetheless the Hexoskin was selected due to its lower cost and garment-based form factor which was deemed more practical for this particular population by the research team (See Supplementary Material). Delsys was selected as the EMG solution, potentially capturing data relating to muscle power, control of voluntary movement and muscle tone, as again it met all the relevant selection criteria around measurement requirements, interoperability, access to raw data and portability. The solution was also clinically validated, medically certified and used with people with CP. Compared to the other solutions investigated, Delsys was found to have slightly lower cost and good availability of software tools (Supplementary Material). Finally, for joint mobility and movement, Xsens was selected as the most appropriate solution. Many of the solutions in this category were targeted at motion capture and were not clinically validated. Xsens had been used with people with CP and had a lower price compared to the other solutions evaluated (Supplementary Material).

Other user-prioritised domains, such as pain, activity and participation, health-related quality of life, ease of care and self-concept, could not be mapped to sensor technology. These are nonetheless important to users and will be captured through validated questionnaires.

## Discussion

The process reported here provides detailed insights to support decision-making when developing the Digital Toolkit, a sensor-based solution for evaluating movement in children with CP, that will be used in future clinical research projects. Through rapid review of the current evidence base and engagement with healthcare professionals, children and families, and industry, a broad range of perspectives were obtained on the domains that should be considered. This was followed by a systematic selection and evaluation process for sensor technologies that addressed the prioritised domains.

Use of pre-identified core outcome sets for CP (19), further, refined by deliberative workshops with stakeholders, allowed the research team to quickly identify meaningful domains of importance for potential inclusion in our Digital Toolkit, such as sensation of pain, exercise tolerance, joint mobility, muscle power, muscle tone, and control of voluntary movement. Interestingly, although families and clinicians had differing views on the relative importance of a number of prioritised domains, there was significant agreement between stakeholders on domains considered to be less important. These included weight maintenance, respiratory function, involuntary movement, gait pattern, and muscle endurance. This is perhaps unsurprising as therapeutic walking devices typically aim to provide opportunities for upright mobility, weight-bearing through the legs and changes in posture, as opposed to delivering exercise at an intensity sufficient to build muscle endurance or alter respiratory function and weight. Clinicians and families also agreed that outcomes related to health-related quality of life, ease of care, and self-concept were important to consider when evaluating movement related to mobility devices. Although, these outcomes do not correspond to domains listed in the ICF-CY core set for CP, they are important to consider when introducing new devices or interventions in line with family-centred care principles. Findings from the deliberative workshops with stakeholders directly informed the subsequent device search and device evaluation and ensured that the Digital Toolkit will be useful and relevant to families, clinicians, and industry.

When mapping sensor technologies in **Step 2** of the process reported here, we identified commercially available, instrumented measures for all the prioritised domains, except for sensation of pain. In the 2020 updated definition of pain, the International Association for the Study of Pain acknowledge the nuances and complexity of pain, and the difficulties in assessment of this sensory and emotional experience (33). Even though pain is very common, given that it is a personal experience influenced to varying degrees by biological, psychological, and social factors, the lack of sensor-based measurement solutions is not surprising. Our device search also highlighted that for some domains, such as muscle power and control of voluntary movement, instrumented measures are not yet well-established and remain subject to ongoing research and validation (24), whereas, devices such as sEMG and MoCap are used more commonly with our population of interest. A potential challenge with some of the identified devices was the ability to collect data in “real-world” settings, i.e., outside of a laboratory environment.

A key requirement in developing our Digital Toolkit was portability as in future studies we plan to evaluate the effects of mobility devices in “real-world” settings, rather than in a laboratory environment. Wearable technologies, particularly garment-based solutions, were noted as having huge potential in the monitoring of movement outside of the laboratory setting. Garment-based solutions which incorporate textile electrodes are available for vital signs monitoring and EMG. Benefits include comfort (over the application and removal of traditional adhesive sensors). However, incorporating sensors within a garment also raises challenges for researchers in relation to the need for multiple sizes (particularly when working with children), cleaning regimens, and loss of precision in sensor placement. The issue of sizing was also apparent in vital signs monitoring devices, such as chest straps, with few paediatric sized products being available, especially for younger children.

Variation in measurement precision was noted when evaluating various vital signs monitors, EMG systems and 3D motion capture systems. Typically, devices could be categorised as (1) research/clinical grade, or (2) consumer grade solutions. For example, for sEMG, full body solutions such as Delsys have been clinically validated and used in a range of populations in a research setting, whereas, the more “consumer grade” Myontec Mbody 3 EMG shorts do not appear to be clinically validated. A similar situation was identified for 3D motion capture when comparing the Xsens Awinda system to the lower cost Shadow MoCap. Whilst these consumer grade systems are less costly (i.e., ~£12,000 for Xsens vs. £900 for Myontec) it is difficult to accurately compare data quality and precision without a formal validation study. Given that our Digital Toolkit will be used with children with CP, where changes in outcome measurements can be small yet clinically meaningful, we selected only solutions which had been clinically validated or used extensively in research as specified in our Device Evaluation selection criteria (Methods, **Step 3**).

From an interoperability perspective many of the devices were found to have either an SDK or API. This allows good access to data in its rawest form whilst also allowing for integration of multiple systems. Integration and synchronisation will be one of the key technical challenges with data processing associated with our Digital Toolkit as it will necessitate combination of data from a number of systems that have different data transmission rates, protocols and data formats. Therefore, when selecting the final set of devices for inclusion in the Digital Toolkit, it was important to consider them together as a system. Selecting a research platform like Shimmer, which provides a number of add on boards to collect a range of physiological and kinematic parameters such as ECG and EMG, may provide flexibility to create a bespoke integrated solution on a single platform. Doing so may, however, have a disadvantage in that technical development and software tools that have already been developed and validated through extensive research cannot be used. Therefore, the research team decided to select established commercial systems (Methods, **Step 2**, Device Selection) that had been validated and provided established software for analysis (Methods, **Step 3**, Device Evaluation).

Review of the device evaluation matrix and consideration of the practicalities of use, measurement precision, interoperability and cost of the various devices, facilitated selection of a final set of sensors for inclusion in the Digital Toolkit that met the priorities of families, clinicians and industry partners. These included the Hexoskin Classic Pro shirt for vital signs monitoring, the Delsys Avanti Trigno for EMG and the Xsens MTw Awinda for 3D motion capture. Combination of these three solutions provide instrumented measures covering all the clinical domains of interest. These products also allowed for integration and synchronisation and were flexible enough to be configured to measure the muscle groups and movements required when evaluating mobility devices. Furthermore, the sensors are wireless, comfortable and can be used outside of a laboratory setting. All the solutions selected have also been clinically validated and used in a paediatric population.

### Reflections and Critical Perspective

#### Comparison to Other Frameworks

To the authors' knowledge, this is the first reported use of previously published frameworks by Booth et al. (16) and Caulfield et al. (12) in the selection and evaluation of sensor technologies for ambulatory human physiological measurement. The work presented combines the criteria for device selection and evaluation from both frameworks and expands upon the frameworks in relation to end-user involvement. The framework presented by Booth et al. (16) assumes that measurement domains have been decided upon and that the research team has performed a device search. It focuses on selecting and managing sensor technology within a research study. The framework presented by Caulfield provides considerations for the device search and criteria for selection of devices, however, the user involvement is limited, and the focus is mainly on technical and human factors (12). A summary of the user involvement in the current study, as per the GRIPP2, is reported in Table 4. The workflow presented in the current report is summarised in Table 5, which also compares items between the frameworks. In relation to defining requirements, the current work expands on the previous frameworks by increasing the focus on identification of the priorities for measurement through engagement with end users.

**Table 4:** GRIPP2 short form.

**Item**	**Application in current study**
**1: Aim** Report the aim of PPI in the study.	•To obtain a broad range of perspectives from end-users on the relative importance of the short-listed domains of interest for potential inclusion in a Digital Toolkit.
**2: Methods** Provide a clear description of the methods used for PPI in the study.	•ICF-CY domains and their associated definitions, as identified through rapid review, were presented in separate workshops to three stakeholder groups: (i) children with cerebral palsy and their families; (ii) clinicians; and (iii) industry. •The workshops were facilitated by both the clinical and computer science researchers. •A high-level summary of findings was communicated directly to all workshop attendees and circulated *via* the Northern Ireland Cerebral Palsy Community Mailing List to disseminate the findings to the wider community.
**3: Study results** Outcomes – Report the results of the PPI in the study, including both positive and negative outcomes.	•Similarities and differences were identified between groups of stakeholders regarding domains they considered important to measure. •Domains common to both families and clinicians were mobility of joint functions and sensation of pain. •Families' top-ranked domain was muscle tone [ranked 7th (of 11) by clinicians]. •Clinicians' top-ranked domain was exercise tolerance [ranked 9th (of 11) by families]. •With regards activity, only “moving around using equipment” was common to both respondent groups. •Strong agreement between clinicians and families was evident in relation to the importance of health-related quality of life, ease of care, and self-concept. •Domains rated least important were broadly consistent between stakeholder groups (and thus removed from subsequent steps). •No further feedback was received from attendees or the wider community in response to disseminated results.
**4: Discussion and conclusions** Outcomes – Comment on the extent to which PPI influenced the study overall. Describe positive and negative effects.	•The process identified meaningful domains of importance for potential inclusion in our Digital Toolkit. •Findings from the user engagement workshops directly informed the subsequent device search and device evaluation and ensured that the Digital Toolkit will be useful and relevant to families and clinicians.
**5: Reflections/critical perspective** Comment critically on the study, reflecting on the things that went well and those that did not, so others can learn from this experience.	*Strengths* •Co-facilitation of the user engagement workshops ensured that all members of the team shared a common vision and language with families and healthcare professionals. •Workshops methods were tailored according to the stakeholder group. *Limitations* •Some families were unable to attend workshops due to work and family commitments. •Further engagement after the communication of the results could have been used to confirm the domains of interest.

*PPI, patient and public involvement*.

**Table 5:** Comparison of frameworks.

		**Booth et al. (16)**	**Caulfield et al. (12)**	**Current report**	**Planned work**
Requirements definition	Identification of potential domains of interest			✓	
	Rapid review of currently used outcome measures			✓	
	User engagement to refine domains of interest			✓	
	Observation of user in scenario in which sensors are intended to be used				✓
	Consideration of human and technical factors		✓		✓
Device search	Mapping of domains of interest to potential sensors		✓	✓	
Device evaluation	Evaluation and selection of sensors	✓	✓	✓	

#### Strengths

The multidisciplinary approach utilised throughout the described process was integral for developing a common sense of purpose within the team. Both clinical researchers and computer scientists met regularly to discuss and prioritise tasks, share work in-progress and were involved in decision-making steps. Co-facilitation of the deliberative workshops with end users ensured that all members of the team shared a common vision and language with families and healthcare professionals.

Workshops were tailored according to the stakeholder group. For example, in the healthcare professional workshop, individuals were asked to consider their responses independently to avoid deferential effects and inhibition of responses within a group of mixed seniority, whereas, the patient and family workshops took a more informal and collaborative approach in which discussion was encouraged (34). This allowed the research team to gain more insight into the differing perspectives within the group. The rapid review and user engagement methods described expand on the processes described by Caulfield et al. (12) and Booth et al. (16) which have a greater focus on the later phases of device selection and evaluation (see Table 5).

#### Limitations

Rapid reviews have the advantage of being time efficient however they have some inherent limitations in comparison to a systematic review process. In the case of this report, one reviewer performed study selection and data extraction, and older articles, non-English articles and grey literature were not considered.

With respect to the deliberative workshops, many families showed an interest and motivation to engage. However, despite having various time slots available, some families were unable to attend due to work and family commitments. This would suggest that creative strategies to involve families and individuals with CP would be of benefit. Strategies that do not involve travel, that remove the need to arrange time away from work, or the need to arrange childcare may open opportunities to others.

Further, user engagement after the communication of the results of the workshops could have been used to confirm the domains of interest. However, by including all four top ranked domains from both the family and healthcare professional workshops it is likely that most user requirements have been captured.

From a technical perspective, the sensors selected incur a substantial purchasing cost with the estimated total price exceeding £25,000. Whilst this represents a significant outlay, and may preclude roll out of the Digital Toolkit in routine clinical practice, it is hoped that findings from development of the Digital Toolkit and collection and interpretation of data will inform future research directions such as investigation of a minimum set of sensors that can be utilised and development of a lower cost solution for longitudinal monitoring in a community setting.

Finally, in relation to scope of purpose, the development of the Digital Toolkit was focussed on how best to assess the effect of mobility devices in children with CP. If the scope of purpose were to be expanded, e.g., to other rehabilitative and assistive devices, repetition of all three steps in the reported process should be undertaken.

### Future Work

Further evaluation of the Digital Toolkit is planned. Future work will focus on feasibility of measuring the chosen domains with the selected sensor technologies in a group of children with CP. In brief, the objectives will be to assess fidelity and safety of testing procedures, to record any technical issues with the sensors, to explore the sensors' ability to discern between two movement tasks, and to use qualitative methods to gain an understanding of the perceptions and preferences of children and families with CP in relation to the sensor technologies employed.

## Conclusion

This report demonstrates a systematic approach to user engagement, and device selection and evaluation, during the development of a sensor-based solution to a healthcare problem, building on previous frameworks. It also provides a narrative on the benefits of employing a multidisciplinary approach throughout the process. The approach resulted in the selection of valid, reliable, commercially available, interoperable sensors that address the priorities of families, healthcare professionals, industry partners, and academic researchers when evaluating the effects of mobility devices on movement of children with CP.

## Data Availability Statement

The original contributions presented in the study are included in the article/Supplementary Material, further inquiries can be directed to the corresponding author/s.

## Ethics Statement

The Research Governance Office in Queen's University Belfast confirmed that ethical approval was not necessary for the presented project.

## Author's Note

In this article we present a systematic process to guide the multidisciplinary team through user engagement and sensor device selection and evaluation. Our approach builds on those previously reported in the literature and expands on the user engagement phase. It also provides a ‘use case' where clinical researchers and computer science researchers collaborate to develop a Digital Toolkit for the assessment of movement in children with cerebral palsy.

This report provides much needed guidance on how to facilitate multidisciplinary collaboration and has an emphasis on patient experience and user engagement in the field of Digital Health.

## Author Contributions

All authors listed have made a substantial, direct and intellectual contribution to the work, and approved it for publication.

## Conflict of Interest

LF is an employee of Sunrise Medical and contributed to the conception of the project but was not involved in the user engagement or device search and evaluation. The remaining authors declare that the research was conducted in the absence of any commercial or financial relationships that could be construed as a potential conflict of interest.
